# Synapse: A co-designed neurodivergent peer support programme for higher education settings

**DOI:** 10.1177/13623613251320448

**Published:** 2025-02-24

**Authors:** Aisling Coyle, Liam O’Hare, Devon Ramey

**Affiliations:** Queen’s University Belfast, Belfast, UK

**Keywords:** co-design study, connectedness, empowerment, higher education, neurodiversity, specialist peer mentoring programme

## Abstract

Transitioning to higher education can be challenging for neurodivergent students and they can be particularly vulnerable to experiencing stress in this new environment, resulting in higher levels of non-completion. To address this issue, this study details the co-design of a support programme for neurodivergent students in higher education. It used logic modelling workshop methodology to identify the short-term and long-term outcomes, activities and resources required for implementation. The logic model was produced during a workshop that involved a range of stakeholders, including researchers, neurodivergent and neurotypical students and disability service staff at an Irish university. The qualitative data were analysed through thematic analysis and three key outcomes were generated from the logic modelling process: ‘connectedness’, ‘knowledge and awareness of neurodiversity’ and ‘empowerment’. The main activities desired by stakeholders included one-to-one mentoring and group-based mentoring, which would allow neurodivergent students to establish new friendships and develop a sense of belonging within the university. The implementation factors identified were providing a dedicated space and mentor pairing. A key finding was that stakeholders did not favour traditional peer mentoring labels of ‘mentor’ and ‘mentee’. Instead, students preferred the development of a symbiotic co-mentoring relationship between peers, with both participants being referred to as ‘mentors’.

**Lay abstract:**

Neurodivergent students may require support with the social aspects of university life. Peer mentoring describes a relationship where a more experienced student helps a less experienced student by providing advice, support and knowledge. It is an effective way to support students’ transition to higher education. This study involved a wide range of stakeholders including neurodivergent students, neurotypical students, disability service staff and researchers in the design of a peer mentoring programme called *Synapse*. A visual representation of the proposed programme (i.e. logic model) was produced during a workshop with the stakeholders. This visual showed the key outcomes or aims of the programme that was agreed upon by the stakeholders. The workshop was audio recorded and the transcripts were analysed to highlight the main conversation themes. Stakeholders stated that neurodivergent students wanted to feel more connected to others at the university to develop a sense of belonging, they also wanted people to learn more about neurodiversity to reduce stigma and finally, they wanted to feel empowered to take control of their lives and have a voice in decision-making. The two preferred activities of the programme were one-to-one and group mentoring. Uniquely, the participants were concerned with traditional terminology around peer mentoring, in particular the terms mentor and mentee, as it inferred an unequal power dynamic in a relationship. The stakeholders believed that students in the *Synapse* programme should be valued and treated equally, regardless of diagnosis or experience. Therefore, all members of the *Synapse* programme were paired within a co-mentoring relationship.

## Introduction

### Challenges for neurodivergent students within higher education

Starting university is a psychologically challenging time for students because it is a time of transition. There is increased independence, academic demands and the social challenges of forming new relationships (Scott & Sedgewick, 2021; Van Hees et al., 2015). To succeed in higher education without accommodations, students need to be ‘hyperliterate, reasonably numerate, depending on the subject; able to sit still, remember, and concentrate for sustained periods; and able to engage in free-flowing social communication’ (Fung, 2021, p. 176). Both neurotypical and neurodivergent students can struggle with these aspects of higher education; but neurodivergent students can experience more difficulties because of fundamental differences in their development (Fung, 2021).

In a systematic review of neurodivergent students’ experiences in higher education, anxiety accompanied personal, social and academic aspects of their studies (Clouder et al., 2020). The emotional and social demands of university life are common causes of anxiety for autistic students, and it can adversely impact their ability to concentrate and persevere with their studies (Ward & Webster, 2018). Although autistic students have similar perspectives to neurotypical students with regard to initiating and establishing relationships at university (Lei & Russell, 2021), autistic students can feel anxious when interacting with others and are inclined to isolate themselves from peers despite a strong desire to make friends (Gurbuz et al., 2019). This has resulted in lower retention rates for autistic students and other students with disabilities within the United Kingdom (Cage & McManemy, 2022; Gurbuz et al., 2019) and the United States (Herbert et al., 2014; Newman et al., 2011).

One contextual factor that contributes to the anxiety and isolation of neurodivergent students is the ‘hidden curriculum’ – the unwritten institutional expectations of how a student will behave, study or interact (Hamilton & Petty, 2023, p. 2). The hidden curriculum is subjective and situational, as the learning experiences and social relationships within education can vary depending on the institution and the students enrolled (Rossouw & Frick, 2023). Neurodivergent students can have more difficulty adapting to these situations and understanding the abstractions of the social world (Beck Wells, 2022; Byrne, 2022; Crooke & Winner, 2022). Navigating the hidden curriculum at university is a process of trial and error that often results in error for neurodivergent students, which further leads to isolation and emotional distress (Byrne, 2022).

Sensory processing differences are also common in neurodivergent students, and they do not go away in tertiary education settings (Waisman et al., 2022). The university environment can comprise competing sensory inputs such as bright lights and loud gathering areas. Educators can underestimate these sensory challenges, which can further lead to stress and anxiety for neurodivergent students and their inability to cope with the day-to-day challenges of university life (Knott & Taylor, 2014; Robertson & Simmons, 2013). As opportunities for socialisation often involve busy, noisy areas such as canteens, cafes or night clubs, the sensory input from these situations may lead to exhaustion and burnout (i.e. listening fatigue; McGarrigle & Mattys, 2023). This can disincentivise engagement in social activities that promote well-being and reduce isolation.

At university, disability identity issues can further create problems for neurodivergent students due to societal stigma, prejudice and marginalisation (Dwyer et al., 2023; Meyer et al., 2015). Students with a diagnosis during primary and secondary education may have experienced isolation, loneliness and feelings of ‘being different’ (Griffin & Pollak, 2009, p. 29). Therefore, neurodivergent students sometimes choose to drop this identity and create a new one with university peers, but this can prevent them from accessing the necessary support (Hamilton & Petty, 2023). As a result, neurodivergent students may mask or camouflage to fit in with a neuro-normative perspective and minimise visibility in social situations (Cage & Troxell-Whitman, 2019; Cassidy et al., 2020; Pryke-Hobbes et al., 2023; Sedgewick et al., 2022). However, both camouflaging and masking are exhausting and can cause mental health issues and an increased risk of suicidality in this group (Cassidy et al., 2018; Shmulsky et al., 2021). Masking can occur at university because neurotypical peers sometimes view students with autism and other learning disabilities with suspicion and hostility, particularly when classroom activities involve graded group work (Shmulsky et al., 2021). One way to overcome this stigma would be to increase knowledge of neurodiversity within the university by having a neurodiversity-friendly campus (Dwyer et al., 2023; Waisman, 2020).

Supporting students with disabilities to develop social connections can contribute to college retention and improve their self-determination (Getzel & Thoma, 2008). Therefore, facilitating and enabling these connections may be a crucial factor for a support programme to improve student well-being. This was shown by Lei et al. (2020), who embedded structured and unstructured social opportunities into a transition to university pilot programme. The autistic students believed that these opportunities were useful in boosting their confidence and preparing them for university. They looked forward to new social opportunities, particularly in the form of clubs and societies. Another common way to assist students in their transition from secondary to higher education is through peer mentoring (Hayman et al., 2022; Lane, 2020; Lyon et al., 2022; Seery et al., 2021). Peer mentoring is a structured and intentional relationship between a mentor and mentee who are matched by a third party, such as a mentoring programme coordinator (Collier, 2023). A peer mentor is ‘an individual who provides support in a variety of ways, such as coaching, counselling or encouragement with goals’ (Nguyen et al., 2019, p. 668). It has been proposed that mentors do not simply provide mentees with advice; rather, problems are often resolved collaboratively, enabling both mentors and mentees to develop (Law, 2013). Mentoring programmes can improve the emotional and behavioural functioning, academic achievement and employability of young people (DuBois et al., 2002). There is also evidence that peer mentoring programmes enhance students’ sense of belonging, satisfaction with the university, engagement and retention rates (Carragher & McGaughey, 2016; Humphrey & Lowe, 2017). More recently, specialist peer mentoring (SPM) programmes have emerged at several large universities across the globe, including the United States, Australia, Canada and the United Kingdom to provide support that is specifically tailored for neurodivergent students.

### SPM programmes

SPM describes a peer mentoring programme targeting students with disabilities, predominantly those with autism. Many SPM programmes are based on the traditional peer mentoring model, where the mentor is usually a neurotypical student, and the mentee is a neurodivergent student (Hillier et al., 2019; Sanders-Gardner, 2025). These mentoring relationships are traditionally dyadic, with the mentor being viewed as a positive role model and having more experience or knowledge than the mentee (Paul et al., 2013). However, it has been argued that recruiting neurotypical students to improve the social integration of a neurodivergent student suggests that the goal is to model neurotypical behaviour (Crompton et al., 2023). This traditional mentorship model may also limit information flow to a one-directional exchange, and mentors are often assigned without careful attention to diversity, equity and inclusion (Friedman et al., 2021). Goerisch et al. (2019) advocated that universities need to reimagine the concept of traditional mentorship, which is rooted in power and hierarchy, into a more democratic, empowering model, whereby the peers are mentoring ‘with’, not ‘at’ or ‘about’ (Goerisch et al., 2019). Equally, Friedman et al. (2021) stated that mentorship should be a more collaborative and nurturing process built on a reciprocal relationship that provides opportunities for symbiotic growth. These points need to be considered in the development of an SPM programme to avoid stigmatisation and improve the inclusivity of neurodivergent students.

### This study

In light of these issues, this study used a co-design approach to develop a peer support programme for higher education settings with both neurodivergent students and neurotypical students, as well as other stakeholders. A logic modelling session guided the development of an SPM programme that was then implemented the following academic year (Coyle et al., 2025). A logic model is a systematic and visual way to present and share understanding of the relationships among the resources required to operate a programme, the activities planned and the changes or outcomes hoped to be achieved. The value of the logic model is in the process of creating, validating and modifying the model over time (Kellogg Foundation, 2004).

In line with previous research on SPM programmes (Ames et al., 2016; Roberts & Birmingham, 2017; Siew et al., 2017; Thompson et al., 2018, 2020), neurotypical students were involved in the development of the support programme as they would be participating in the programme with the neurodivergent students. To meet this aim, the study focused on the following research questions in line with the key aspects of programme development:

1. What outcomes do stakeholders wish to improve through the programme?
2. What programme outputs/activities do stakeholders think will improve the programme outcomes?
3. What implementation factors do stakeholders think should be considered for the programme?

## Method

### Procedures for sample selection and recruitment

Neurodivergent students were recruited through disability services via email. A poster was designed to recruit mentees, and an oral presentation was given to all disciplines in Years 1–3. Neurotypical students were recruited through the university student email, a poster was designed to recruit mentors, and an oral presentation was delivered to all disciplines in Years 1–3. The recruitment presentation outlined the traditional model of peer mentoring and invited the students to take part in the research as co-designers of a peer mentoring programme specifically tailored for neurodivergent students. Students were invited to take part in a workshop and/or the programme, and mentors who participated in the programme would receive a Special Purpose Award-Certificate in Peer Mentoring awarded by the University.

All students (neurotypical and neurodivergent) were invited to participate in the workshop. On the consent form, students were simply asked whether they identified as neurodivergent or not, and they were not required to be registered with disability services. This enabled all students to participate, especially any neurodivergent student who may not have chosen to disclose their neurodivergent condition. Specific data on socioeconomic status and educational attainment levels were not recorded. A memorandum of understanding (MoU) was developed with disability services, which outlined the roles and responsibilities of all stakeholder groups, that is, the student mentors and mentees, disability staff at the university and the research team. A mentor was described as a person who would support a peer mentee by helping them settle into university, through one-to-one meetings and/or group meetings. A mentee was described as a student who received support and guidance from a peer through their first year of university life. The MoU also included the research study time frame. The MoU and informed consent clearly articulated written descriptions of the roles and responsibilities of all stakeholders in the research. In attendance were neurodivergent (*n* = 17) and neurotypical students (*n* = *18*), disability staff (*n* = 3) and researchers (*n* = 2). Two members of the disability team identified as neurodivergent. The researchers included a doctoral student researcher with personal experience of being a mother to a neurodivergent child, and a research supervisor with specialty in the area of social science and education, and experience working with young people as partners in the development of programmes through logic modelling.

### Research design

This study used a qualitative research design, whereby the students and other stakeholders participated in a logic modelling workshop. Co-design has become important in the face of complex educational issues where no one person has the knowledge and skills to understand and solve them, and a new approach was needed to empower people to participate (Zamenopoulos & Alexiou, 2018). Logic modelling was applied from the outset as it provided a roadmap for the development of the programme, including what activities needed to take place and how the desired outcomes were to be achieved. Tools like logic models can increase the participant’s voice in programme design, implementation, analysis and knowledge generation (Kellogg Foundation, 2004). Logic models ensure that programme evaluations focus on the ‘right’ outcomes and test the ‘right’ underlying theories that the programme designers had in mind (Easterling et al., 2023, p. 2).

From an epistemological perspective, this research took the position of an advocacy/participatory paradigm, which concerns those who are disempowered or marginalised and considered to have a lack of voice, thereby bringing the research under the umbrella of critical approaches, which include disability, gender, race or ethnicity (Cohen et al., 2018). Furthermore, it can be argued that human knowledge only captures a portion of a much deeper and more vast reality (Fletcher, 2017), and real knowledge exists behind and within the subjective interpretations people apply to the world (Braun & Clark, 2013). This research fitted the ontological position of critical realism because the qualitative data produced in the co-design session resulted in the development of a programme that was feasible and based on mutually agreed outcomes, as well as being influenced by the rules and hierarchy of the social world.

### Procedure

The logic model was produced during a single workshop at the university. In the workshop, presentations were delivered by the research team and disability services, afterwards stakeholders participated in designing the programme. The presentations were held so that potential participants could make an informed decision about participating in the workshop, and to build capacity to fully participate in the logic modelling activity. In these presentations, the students were introduced to “the term neurodiversity and described the benefits of peer mentoring. In addition, the structure of the SPM programme delivered at the University of Massachusetts was briefly described. Finally, specific details about how to develop a logic model were provided. Logic models are developed from a series of causal statements of programme components and theory. Based on the recommendations made by Connolly et al. (2017), the researcher asked the following questions: What measurable outcomes are desired? What is the pattern of change between these outcomes (i.e. theory of change)? What outputs/activities should be done to achieve these outcomes (i.e. theory of intervention)? What resources are available to carry out the activities (i.e. theory of implementation)? What is the context surrounding the programme (i.e. external factors)? and What needs to be in place for the programme to be adequately implemented (i.e. assumptions)? The stakeholders’ responses were recorded on the whiteboard and the researcher then created the logic model with the stakeholder group. The logic model presented their views in a visual format and demonstrated how each component interacted with the other. The logic model provided a visual representation that explicitly outlined how the programme activities would improve the outcomes of the target group (Connolly et al., 2017).

### Data analysis

The responses to the workshop questions were audio-recorded and reflexive thematic analysis (Braun & Clarke, 2022b) was used to interpret patterns across the audio transcriptions. The philosophical theory underpinning the research was critical realism, as the experiences and understanding of neurodiversity were mediated by the language and culture of the participants, and the researcher aimed to conceptualise their different perspectives for the design of a support programme for neurodivergent students (Braun & Clarke, 2022b).

Author 1 used Otter.ai^®^ software to convert the audio files to text, then manually edited the transcripts by listening to the audio files. Although the analysis was contained within a logic model framework (i.e. outcomes, inputs, outputs, the themes within each section of that framework were not pre-determined. The themes were developed from patterns of shared meaning, united by a central idea, and the codes were developed organically with no use of any codebook (Braun & Clarke, 2022a). Therefore, thematic analysis took an experiential, inductive, semantic approach, whereby the researcher allowed the themes to be produced naturally from the data without preconceived ideas, and the interpretation stayed close to the language of the participants (Braun & Clarke, 2022b). This research aimed to ‘give voice’ to the participants and enabled them to share a glimpse of their perspectives on the support needs of neurodivergent students in higher education (Braun & Clarke, 2021). Sub-themes were generated and collated into three overarching themes that reflected the outcomes the stakeholders desired. Author 1 then met with Author 2 and Author 3 to review and define the identified themes. The design of the support programme was focused on achieving these outcomes and the themes provided explicitness of the programme’s purpose to the participants.

### Community involvement

The study facilitated a workshop that employed a co-design approach due to its meaningful involvement of end-users. Specifically, autistic students and other neurodivergent students participated in a co-creation workshop to develop an SPM programme that was subsequently implemented in the 2022–2023 academic year (Coyle et al., 2025). Two disability staff members identified as neurodivergent, while both researchers were non-autistic and identified as neurotypical. The lead researcher (corresponding author) is the mother of a neurodivergent child. Collectively, this team created a balance between researchers, neurodivergent community leaders and neurodivergent students, which is crucial for participatory co-design and aligns with a crucial aspect of the inclusion of autistic adults as study participants, as outlined by Academic Autism Spectrum Partnership in Research in Education; (AASPIRE) international practice-based guidelines (Nicolaidis et al., 2019).

## Results

The thematic analysis of the workshop transcriptions suggested that social and emotional development was the group’s primary concern, as each theme (i.e. connectedness, knowledge and awareness of neurodiversity and empowerment) aimed to improve the social experiences of the neurodivergent students in higher education. The qualitative findings are presented in narrative form with indicative data extracts to convey the participants’ responses, demonstrating sub-themes and themes related to the three research questions. These narratives were summarised and developed the logic model, which described the programme outcomes, activities and implementation factors.

### What outcomes do stakeholders wish to improve through the programme?

**Figure 1.**
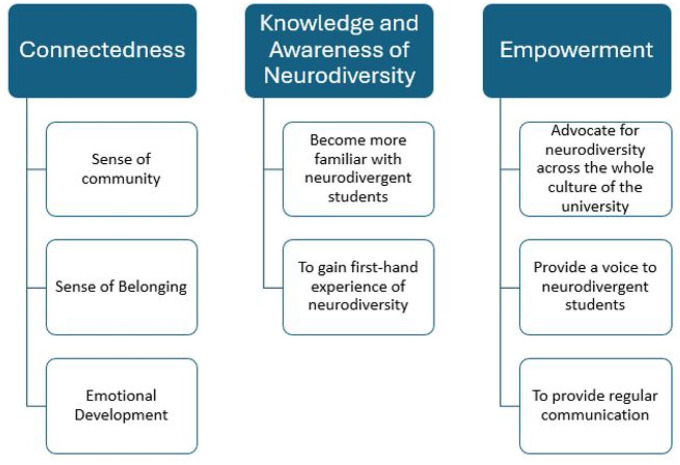
Thematic analyses output map identifying themes and sub-themes of outcomes desired by stakeholders.

Three main themes/outcomes were identified from the workshop data: ‘connectedness’, ‘knowledge and awareness of neurodiversity’ and ‘empowerment’, each comprising several sub-themes (see Figure 1).

#### Connectedness

Connectedness was the first long-term outcome desired by the stakeholders, with three sub-themes identified: ‘sense of community’, ‘sense of belonging’ and ‘emotional development’. First, neurodivergent students stated they wanted a peer support programme that helped them meet and connect to other neurodivergent students, which was exemplified by the following quote:

‘Establishing a sense of community . . . like make it so neurodiverse students can find other neurodiverse students’. (Neurodivergent Student)

‘And a sense of belonging’. (Neurodivergent Student)

Disability staff and management explained that such help was available at the university; however, enabling neurodivergent students to reach out and access support had been difficult. The reasons for this may have been multi-factorial, but the disability team suggested that it may have been due to a lack of social and emotional and/or self-advocacy skills:

‘We already have a lot of academic support in place. But the difficulty is getting the students to come and engage with it; therefore, having the social and emotional skills to be able to make that first step and ask for help is really important’. (Disability Staff Member)

By the end of the workshop, it was clear that stakeholders prioritised the development of social and emotional skills over academic skills. There was a belief that the two interacted, but the group highlighted that being comfortable and emotionally stable was a prerequisite to academic learning.

‘I think social [integration] might be slightly more important than academic because you need the baseline of social for some of the academic things’. (Neurodivergent Student)

#### Knowledge and awareness of neurodiversity

Knowledge and awareness of neurodiversity was the second long-term outcome identified, with two sub-themes: ‘become more familiar with neurodivergent students’, and ‘to gain first-hand experience of neurodiversity’. Neurotypical students from caring disciplines (e.g. education and occupational therapy) wanted to learn about neurodiversity from an altruistic perspective and for future career advancement. Equivalently, neurodivergent students expressed a desire to learn more about neurodiversity from other neurodivergent students as a process of self-discovery. They also hoped that neurotypical peers could learn about neurodiversity by being in the programme.

‘To have a better understanding of how neurodiverse [sic] students think and how they act, because I know well in industry now, diversity is a huge thing. So, you need to know how to work with people who are neurodiverse and having an understanding would be good for employment’. (Neurotypical Student)

‘We are doing a module called Discovering Autism, and it is different seeing it in the real world, but then as well, like for inside your own self, just satisfaction for helping someone else, so that is kind of why I signed up’. (Neurotypical Student)

‘I want more of the staff and lecturers outside of disability services to have a better understanding of neurodiversity’. (Neurodivergent Student)

#### Empowerment

The third long-term outcome identified was empowerment. The three sub-themes were: ‘advocate for neurodiversity across the whole culture of the university’, ‘provide a voice to neurodivergent students’ and ‘provide regular communication’. A positive strengths-based attitude towards neurodivergent students emanated throughout the workshop session, and there was a desire to spread awareness of neurodiversity throughout the entire campus. To clarify this point, a member of the research team proposed the advocacy role of a mentor, and everyone in the workshop agreed with this position.

‘A neurodivergent mentor in this programme can act as an advocate for neurodiversity across the whole culture of the university’. (Researcher)

Regular communication and checking in with neurodivergent students were highlighted as important for preventing dropouts and centring their voices:

Asking the students, you know, the like getting an idea of what’s going on like . . . giving them a voice . . . . . . I guess any particular struggles that would lead to dropping out . . . . . . by assessing progress or some sort of maintenance . . .. Checking in. (Neurodivergent Student)

The lack of neurodiversity awareness among lecturing staff was a prominent issue for the students and it highlighted a need for further education and training, which was identified as a short-term goal of the programme.

‘Some [staff] already have a good understanding, and then others, you know, have never even thought about it’. (Neurodivergent Student)

The stakeholders suggested that progress towards awareness and inclusion could only be achieved by including both sets of individuals in the conversation, reflecting a symbiotic dialectic. Students who attended the workshop to become mentors believed that one could only understand neurodivergent students by being in their company in a relaxed, non-judgemental environment.

### What programme outputs/activities do stakeholders think will improve the programme outcomes?

#### Activities to improve connectedness

The students thought that connectedness within the programme could be achieved through simple group activities, such as playing games or participating in social events together. Playing games was thought of as an activity to reduce pressure to have a conversation and engage in eye contact. These simple interactions were thought to give students time to feel comfortable with other people in the group and provide opportunities to develop social skills.

‘Create a programme where people can socialise by playing games or simply just have an interesting space to hang out . . . for people that aren’t really comfortable socialising . . .so, it kind of leads them into socialising unintentionally’. (Neurodivergent Student)

The stakeholders felt strongly that having one-to-one mentoring in addition to a group session was necessary. Stakeholders described how one-to-one mentoring would allow for discussion of private and personal issues, enabling the students to access appropriate support when required.

‘I feel that the one-to-one is more intimate; people can share what they want to say. Whereas in a big group, people might be more nervous and may not say what they feel like at that moment. So, one-to-one would give them that opportunity’. (Neurodivergent Student)

Including a group session within the programme was important for making multiple social connections. The stakeholders cited ‘safety in numbers’ and having access to a group would ensure that there would be someone to talk to each week when a one-to-one mentoring session may not happen:

‘Have a dedicated room without noises for meeting up’. (Neurodivergent Student)

‘A space where people can socialise, or just an interest space, to play games when they are not really comfortable socialising, so it kind of leads into socialising’. (Neurotypical Student)

#### Activities to improve knowledge and awareness of neurodiversity

To improve knowledge and awareness of neurodiversity, the group suggested that good quality (evidence-informed where possible) information relating to autism, attention deficit hyperactivity disorder (ADHD), dyslexia and dyscalculia be selected, curated and posted on the virtual learning environment Blackboard^®^, as it was the university’s learning management system.

‘Sometimes information on social media can be misleading or not comprehensive . . . good quality information means that students will not run into problems later on’. (Disability Staff Member)

The stakeholders requested several delivery modes (e.g. videos, websites, podcasts and blogs), and suggested that this material could stimulate group discussions on neurodiversity. Peer learning of neurodiversity was seen as crucial to developing knowledge and awareness of neurodiversity:

‘I know a lot of neurodivergent adults, and they like being involved. They do think that the best way to learn about conditions like autism or ADHD is to talk to someone with those conditions. You know you are building your character and experience for your future, going out into the workforce, and going out into the world, and I think quid pro quo’. (Neurotypical Student)

#### Activities to improve empowerment

To improve feelings of empowerment within the programme, the stakeholders suggested that the one-to-one mentor set goals and tasks related to university life. Regular communication through emails or one-to-one meetings would help prevent neurodivergent students from dropping out.

‘[To increase retention] ask the student, you know like to get an idea of what’s going on like . . . give them a voice’. (Neurotypical Student)

The group was eager to invite lecturing staff and future employers to group sessions to promote the unique strengths neurodivergent students can bring to those settings. In addition, the stakeholders were eager to highlight unique challenges for neurodivergent students in university, such as sensory issues and the need for movement breaks, seating arrangements in class and the need for flexibility in relation to assessments.

‘We may have students who drop out because they come into university and they want to do a particular thing . . . then there are modules there that are not related to that thing at all . . . they have no problem in the thing that they are interested in, but then they really struggle with things that they just don’t see as relevant . . . therefore inviting academic staff to the programme may help to understand a neurodivergent students perspective’. (Disability Staff Member)

To improve self-advocacy and self-efficacy skills among the students, a member of the disability team suggested that role-play activities with members of the group or academic staff would be a good activity to develop the social skills needed for future employment:

‘You know what is to say that an autistic adult going into university could benefit from having some scenarios that can be rehearsed before they face those real scenarios, have it in a safe place, and then go into the real world’. (Disability Staff Member)

Taking part in a group social outing was favoured as the ultimate activity to conclude the programme at the end of the semester. However, students needed to feel comfortable with others in the group before taking part in this activity because of apprehension about new social situations.

### What implementation factors do stakeholders think should be considered for the programme?

#### Dropping the label ‘mentee’

The stakeholders wanted to drop traditional peer mentoring terminology, which labels participants as either ‘mentors’ or ‘mentees’. The students suggested that the term ‘mentee’ inferred a power imbalance within the mentoring relationship, with the mentee being seen as the only person who needed help. Students indicated that these terms influenced their decision to participate in the programme and thought they could affect future recruitment of neurodivergent students. One neurodivergent student suggested all participants should be called a mentor and the rest of the group agreed. The students commented:

‘I just think it makes everyone equal’. (Neurodivergent Student)

‘I put my name down for this research as a mentor even though I have ADHD . . . I did not want my classmates to view me as a mentee, who was in need of help’. (Neurodivergent Student)

The researchers and the disability team agreed that everyone enrolled in the programme would be called a mentor, because all involved would mentor each other on life at university while learning about neurodiversity. In this way, the peer relationship would be viewed as a symbiotic partnership between students, regardless of neurotype, where both students could grow and learn together. Dropping the label of the ‘mentee’ was seen as a positive move towards equality and inclusion. In addition, the disability team raised a concern regarding the *Special Purpose Award Certificate in Peer Mentoring*. During recruitment, the award was originally advertised and offered to students who acted as a mentor. For stakeholders, promoting equality and inclusion was an important aspect of the programme; therefore, a member of the disability team suggested:

‘It would be very useful for everybody that they all receive the award at the end’. (Disability Staff Member)

#### Dedicated space for the programme

In the university, dedicated sensory rooms were available, and the disability team said that the students could book these rooms for one-to-one meetings. For group sessions, the students were eager to have a dedicated space for neurodivergent students to facilitate socialisation within the group. The layout of the room was important to the students. One student commented:

‘A common room, focusing on socialising and chilling out; this room here, I love it, couple of round tables, space to chill out and a more comfortable area as well’. (Neurodivergent Student)

#### Mentor pairing

On being matched with a mentor, students wanted options at enrolment, such as being matched by discipline or particular interests where possible, followed by random assignment.

‘I think it would be better to match up with the course because if there are questions about the course and you’re doing the same course, you would have more information about it’. (Neurotypical Student)

‘I think by interest would be a good idea’. (Neurodivergent Student)

‘I think it should be a bit of a mix, give options’. (Neurodivergent Student)

The group collectively felt this approach was acceptable. Some students were apprehensive about mentor pairing in case the students were incompatible or if one student felt uncomfortable. Therefore, it was suggested that programme coordinators check-in and review mentor relationships three weeks after programme commencement.

## Discussion

This study co-designed a peer support programme for neurodivergent students in higher education using logic modelling. Stakeholders identified three outcomes, which collectively related to the students’ social and emotional development. Two main activities were proposed to improve these outcomes: one-to-one mentoring to provide personalised support, and larger group mentoring sessions to maximise neurodivergent students’ opportunities for socialisation within the university. A major implementation factor that emerged was the desire to drop the traditional peer mentoring labels of ‘mentor’ and ‘mentee’ in favour of a co-mentoring partnership based on a symbiotic mutual relationship. Stakeholders raised concerns about what they considered to be an imbalance of power in mentoring relationships. This was an original finding as compared to previous studies (Ames et al., 2016; Locke et al., 2024; Roberts & Birmingham, 2017; Siew et al., 2017; Thompson et al., 2018) on the topic.

### Creating a programme logic model: *Synapse*

**Figure 2.**
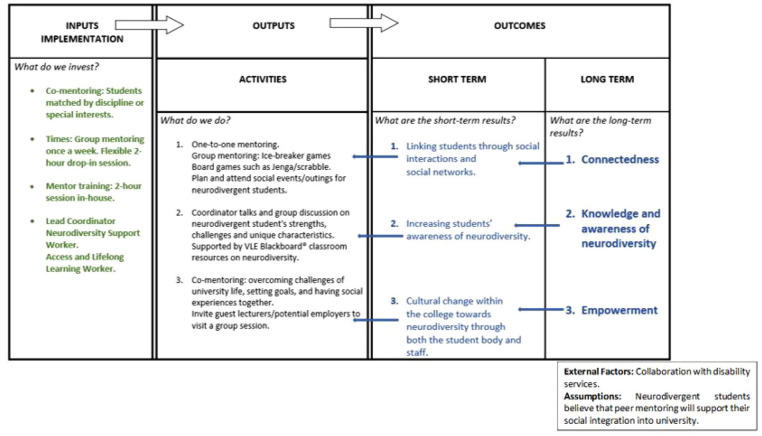
Co-design logic model of a peer mentoring support programme for neurodivergent students, detailing the outcomes (long- and short-term), outputs and inputs necessary.

Looking across the findings, it was possible to use the information to produce a logic model for a neurodivergent peer support programme for higher education settings. The programme was named *Synapse* by the stakeholders during the workshop. The name represented the main outcomes of the programme, which were to establish connections, to increase knowledge and awareness of neurodiversity, and provide a sense of empowerment for the participants. The logic model summarises the main aspects of the *Synapse* programme, that is, outcomes, activities, inputs and implementation factors (see Figure 2). Each of these main programme components are discussed in more detail in the following sections.

### Outcomes

In higher education, peer mentoring programmes have typically emphasised improved academic performance (Collier, 2015); however, this study found that social integration was more important than academic integration to the stakeholders. Consistent with previous research on neurodivergent students, this study found that supportive social relationships are crucial for success in higher education (Bolourian et al., 2018; Cai & Richdale, 2016; Clouder et al., 2020; Gillespie-Lynch et al., 2017; Lei et al., 2020; Lei & Russell, 2021; McPeake et al., 2023; Siew et al., 2017), and that poor social integration can be a major contributor towards feelings of isolation and loneliness (Lei et al., 2020). These findings were reflected in the first theme of connectedness.

Neurodivergent students within the group expressed a strong desire to meet other neurodivergent students to feel more comfortable at university. In fact, there was a sense of urgency in the conversation about finding opportunities to connect with other like-minded people. Research on autistic students’ experiences in higher education has suggested that neurodivergent students who struggle with social interactions have more of a desire to feel connected to others with similar experiences (Buckley et al., 2021; Crompton et al., 2023; Locke et al., 2024; McPeake et al., 2023). In studies where autistic students found like-minded people in support programmes, the process was described as revelatory and transformative (Buckley et al., 2021; Manett, 2022). In a sense, by finding their ‘tribe’, neurodivergent students were more able to find their place in society (Silberman, 2015). Neurodivergent people are often forced to live in a world that is not accepting of their differences and expected to conform to neurotypical behaviours in social interactions, which can cause stress and anxiety (Cooper, 2023). For neurodivergent students to achieve a sense of community, they need others who are accepting of behaviours that default from a neuro-normative perspective (e.g. hand flapping, stimming, averted eye contact; Kapp et al., 2019). Van Hees et al. (2015) noted that autistic students found peers at university more accepting and friendlier than those in secondary education. Therefore, university may present a unique opportunity for neurodivergent students to develop positive social connections that could be supported through this programme.

The second outcome of improving knowledge and awareness of neurodiversity was developed from the perspectives of both neurotypical and neurodivergent students. Neurotypical students’ desire for mentoring was primarily altruistic; however, there was also a strong desire to learn about neurodiversity for personal and career development. Previous SPM programmes have reported that neurotypical mentors found the mentoring experience rewarding and it facilitated networking within institutions, which deepened their sense of belonging (Hamilton et al., 2016; Hillier et al., 2019; Seery et al., 2021). In addition, Buckley et al. (2021) found that the non-autistic mentors gained knowledge about autism, which was a constructive experience that could be applied in their professional lives. On the contrary, neurodivergent students in this study were eager to share their lived experiences with peers and academic staff due to previous feelings of being misunderstood in educational settings. Considering both perspectives, it can be said that these desires reflected the double empathy problem (Milton, 2012), which suggests that both autistic people and non-autistic people can struggle to process and understand each other due to differences in experiencing the world. It was admirable that the neurodivergent students were willing to share their lived experiences. It is recognised that information on lived experience is valuable for the programme design (O’Leary & Tsui, 2022).

Driving and sustaining a cultural change towards neurodiversity awareness across the university emerged in the discussion, and the final outcome of empowerment was developed. The stakeholders recognised that empowerment required scaffolding through activities, which would first develop connectedness and knowledge of neurodiversity, and eventually lead to self-advocacy and retention. This pathway to self-advocacy has been described by Locke et al. (2024), who found self-advocacy skills developed after students made connections within a peer group, which then enabled them to initiate their own study groups or groups based on areas of interest. In a similar way, Lei and Russell (2021) outlined the importance of developing autonomy in autistic students at university, by having the confidence to take responsibility in solving problems and by finding a sense of purpose during this period of transition and increased independence.

The stakeholders in this study also highlighted emotional development as an aspect contributing to the empowerment of neurodivergent students. Disability staff members believed that neurodivergent students often struggled to connect with disability services due to a lack of social and emotional skills. This finding parallels research on post-diagnostic support services for autistic adults, which showed ambivalence among the autistic community with regard to seeking support and often indicated a preference for autistic-led support (Beresford et al., 2020; Coleman-Fountain, & Beresford, 2018; Norris et al., 2025). There is evidence to suggest that neurodivergent students experience more stress, loneliness and anxiety in university as compared to neurotypical students, which can impact their learning and success (Clouder et al., 2020). However, autistic students and students with ADHD can have difficulty identifying emotions, which can affect their relationships and how they understand themselves and the world around them (Guldberg, 2020). Hamilton and Petty (2023) suggest that neurodivergent students need access to welcoming and safe spaces that are non-judgemental in order to develop enjoyable and compassionate memories of university life. This perspective was supported by Siew et al. (2017), who found that autistic students in a mentoring programme appreciated having a mentor when approaching university staff members, as it reduced communication apprehension. The stakeholders of this study further emphasised the need for regular communication and check-ins with neurodivergent students to ensure support and prevent attrition. There was a consensus that if neurodivergent students do not have the opportunity to make friends at university and do not feel comfortable talking to staff, feelings of social isolation could develop and drop-out would be more likely. Previous studies have shown that more frequent check-ins and meetings have contributed to the success of peer mentoring programmes with autistic students (Hillier et al., 2019; Roberts & Birmingham, 2017).

On the contrary, Kim and Crowley (2021) discovered that autistic students with individualised education plans (IEPs) in prior education desired the opportunity for a more ‘normal’ university experience and a chance to become more independent, thereby choosing not to disclose their disability. Disability identity issues are commonly discussed in the literature, whereby students with disabilities who experienced stigma or marginalisation in prior education have opted for a new social identity in higher education (Shmulsky et al., 2021; Van Hees et al., 2018; Ward & Webster, 2018). Therefore, the lack of engagement with disability services by neurodivergent students may be multifactorial. Thus, a successful peer support programme should focus on improving the social and emotional skills needed to reach out for help, establish connections among peers and reduce disability stigma by improving neurodiversity knowledge and awareness within the wider university community.

### Outputs (activities)

The activities stakeholders thought would develop the social and emotional skills were one-to-one mentoring and group mentoring sessions. Traditional peer mentoring programmes have included one-to-one sessions, primarily pairing first-year undergraduate students with more experienced peers (Collier, 2023). However, due to the idiosyncratic nature of neurodivergence among different students, some SPM programmes have offered group sessions to promote socialisation and support (Ames et al., 2016; Locke et al., 2024; Roberts & Birmingham, 2017; Siew et al., 2017). Stakeholders desired both structured (co-ordinator led) activities to learn about neurodiversity and unstructured social experiences; ‘*a space where people can socialise . . . when they are not really comfortable socialising . . . so it kind of leads to socialising*’. Evaluations of SPM programmes that delivered group mentoring sessions on a monthly rather than weekly basis showed that autistic students requested more opportunities to socialise with other autistic students (Ames et al., 2016; Roberts & Birmingham, 2017). Therefore, the activities desired by the stakeholders in this study reflect the approaches taken by many other SPM programmes (Ames et al., 2016; Siew et al., 2017; Thompson et al., 2020). This suggested that a combination of one-to-one and group mentoring activities would provide the strongest support for social integration into the university.

### Inputs and implementation factors

A unique finding of this study was the stakeholders’ attitudes towards the terminology associated with traditional peer mentoring, specifically the labels of ‘mentors’ and ‘mentees’ (Collier, 2023; Goerisch et al., 2019). The traditional model privileged certain forms of knowledge, with the mentor being a more experienced undergraduate or graduate student (Hillier et al., 2019; Roberts & Birmingham, 2017; Siew et al., 2017; Thompson et al., 2020). In these studies, the mentors were recruited to guide, advise and support an inexperienced neurodivergent student. The recruitment posters for this study reflected this narrative. Therefore, this led to initial bias in this study, and it is likely the participants’ first impressions of their potential role in the programme were affected, it is important to recognise underlying assumptions in the literature (Cottrell, 2023). The stakeholders of this study challenged this view by stating the label ‘mentee’ would portray them as inferior or lacking self-efficacy. It would also suggest that the relationship was one-directional with an unequal power dynamic. Neurodivergent students may have prior educational experiences whereby they felt like they were not ‘good enough’, or they have been punished for behaviours which defaulted from the norm (Anderson et al., 2018, 2020; Cai & Richdale, 2016). The societal stigma towards individuals with a disability may have promoted these feelings of inferiority (Hamilton & Petty, 2023). Promoting equality within the programme was important; therefore, the consensus was to abandon the term ‘mentee’ in favour of all participants being called ‘mentors’. To date, no studies have moved away from mentor–mentee terminology in SPM programmes. However, Goerisch et al. (2019) challenged the traditional peer mentoring structure in favour of a more empowering model, which values inclusivity of experience, knowledge and reciprocity. Therefore, the programme would be designed so that all participants could get accredited with a Special Purpose Award Certificate in Peer Mentoring. By creating a co-mentoring reciprocal partnership, the programme could promote equality of lived experience among all students and align better with the social model of disability, thus creating a co-mentoring symbiotic partnership.

Finally, students were eager for the programme to have a dedicated safe space within the university to meet up. This was shared with many other SPM programmes, where it was found that having a safe space for autistic or neurodivergent students to socialise was a crucial element in the development of social networks (Ames et al., 2016; Lucas & James, 2018; Thompson et al., 2020). As this study aimed to develop a programme within one university, a dedicated space was located within the disability services area. It was quiet, airy and had large windows and round tables to stimulate conversations. Having such spaces within a university is key to building neurodiversity-friendly campuses (Dwyer et al., 2023). Dwyer et al. (2023) emphasised the importance of establishing disability cultural centres that are sensory attentive to promote positive disability identities, culture and community.

### Limitations and future directions

It is important to recognise that stakeholders consulted in this study constituted a particular context of one university in Ireland (Atkins & Wallace, 2012). Another potential limitation is that some of the stakeholders may have had difficulty speaking out in the group setting. Smaller groups of similar students (e.g. autistic students) or individual interviews may have uncovered more honest accounts of the difficulties experienced at university. Furthermore, the lead author of this study was a neurotypical researcher who may have failed to fully understand and engage with the neurodivergent students’ perspectives due to the double empathy problem (Milton, 2012). This study did not involve the neurodivergent students in the analysis of the data or production of the research findings. Future research should consider embedding the perspectives of neurodivergent researchers in all stages of the process through community-based participatory research (Nicolaidis et al., 2019).

## Conclusion

This study outlined a logic modelling workshop that was conducted with key stakeholders, including neurodivergent students, neurotypical students, disability support staff and researchers. The logic modelling session guided the development of an SPM programme that was subsequently implemented in the following academic year (Coyle et al., 2025). This study contributed to a growing body of literature on SPM programmes designed for neurodivergent students in higher education by including stakeholders’ views on the development of such a support programme. Three outcomes were identified, which related to improving connectedness, knowledge and awareness of neurodiversity and feelings of empowerment. Neurodivergent students expressed a strong desire to find other neurodivergent students at university and to have opportunities to socialise in safe, non-judgemental environments. Therefore, improving social connectedness should be a key recommendation for future peer support programmes for this group. One unique finding was that peer-mentoring relationships can have unequal power dynamics, which can be offensive to neurodivergent students. Issues arose around the term ‘mentee’, as it indicated an inferior position rather than a symbiotic partnership that should be developed. This more reciprocal approach to peer mentorship could be more empowering for neurodivergent students. The logic modelling session guided the development of an SPM programme that was implemented the following academic year.
